# Effectiveness of Chinese Herbal Medicine as a Complementary Treatment for Neutropenia Prevention and Immunity Modulation During Chemotherapy in Patients With Breast Cancer: Protocol for a Real-World Pragmatic Clinical Trial

**DOI:** 10.2196/55662

**Published:** 2024-03-11

**Authors:** Kai-Hung Wang, Hsuan-Shu Shen, Sung-Chao Chu, Tso-Fu Wang, Ching-Wei Lin, Wei-Han Huang, Yi-Feng Wu, Ching-Chun Ho, Cheng-Yoong Pang, Chi-Cheng Li

**Affiliations:** 1 Department of Medical Research Hualien Tzu Chi Hospital Buddhist Tzu Chi Medical Foundation Hualien Taiwan; 2 Department of Chinese Medicine Hualien Tzu Chi Hospital Buddhist Tzu Chi Medical Foundation Hualien Taiwan; 3 School of Post-Baccalaureate Chinese Medicine Tzu Chi University Buddhist Tzu Chi Medical Foundation Hualien Taiwan; 4 Sports Medicine Center Hualien Tzu Chi Hospital Buddhist Tzu Chi Medical Foundation Hualien Taiwan; 5 Department of Hematology and Oncology Hualien Tzu Chi Hospital Buddhist Tzu Chi Medical Foundation Hualien Taiwan; 6 School of Medicine Tzu Chi University Buddhist Tzu Chi Medical Foundation Hualien Taiwan; 7 Department of Clinical Pathology Hualien Tzu Chi Hospital Buddhist Tzu Chi Medical Foundation Hualien Taiwan; 8 Department of Surgery Hualien Tzu Chi Hospital Buddhist Tzu Chi Medical Foundation Hualien Taiwan; 9 Institute of Medical Sciences Tzu Chi University Buddhist Tzu Chi Medical Foundation Hualien Taiwan; 10 Center of Stem Cell and Precision Medicine Hualien Tzu Chi Hospital Buddhist Tzu Chi Medical Foundation Hualien Taiwan

**Keywords:** complementary treatment for cancer, neutropenia, real-world study, bedside to bench study, immune cell profile, programmed cell death protein 1, PD-1, breast cancer, breast, cancer, oncology, Chinese medicine, herb, herbs, herbal, complementary, immunity, immunology, immunomodulation, immunological, neutrophil, chemotherapy, blood cell, blood cells

## Abstract

**Background:**

In recent years, advancements in cancer treatment have enabled cancer cell inhibition, leading to improved patient outcomes. However, the side effects of chemotherapy, especially leukopenia, impact patients’ ability to tolerate their treatments and affect their quality of life. Traditional Chinese medicine is thought to provide complementary cancer treatment to improve the quality of life and prolong survival time among patients with cancer.

**Objective:**

This study aims to evaluate the effectiveness of Chinese herbal medicine (CHM) as a complementary treatment for neutropenia prevention and immunity modulation during chemotherapy in patients with breast cancer.

**Methods:**

We will conduct a real-world pragmatic clinical trial to evaluate the effectiveness of CHM as a supplementary therapy to prevent neutropenia in patients with breast cancer undergoing chemotherapy. Patients will be classified into CHM or non-CHM groups based on whether they received CHM during chemotherapy. Using generalized estimating equations or repeated measures ANOVA, we will assess differences in white blood cell counts, absolute neutrophil counts, immune cells, and programmed cell death protein 1 (PD-1) expression levels between the 2 groups.

**Results:**

This study was approved by the research ethics committee of Hualien Tzu Chi Hospital (IRB 110-168-A). The enrollment process began in September 2021 and will stop in December 2024. A total of 140 patients will be recruited. Data cleaning and analysis are expected to finish in the middle of 2025.

**Conclusions:**

Traditional Chinese medicine is the most commonly used complementary medicine, and it has been reported to significantly alleviate chemotherapy-related side effects. This study’s findings may contribute to developing effective interventions targeting chemotherapy-related neutropenia among patients with breast cancer in clinical practice.

**Trial Registration:**

International Traditional Medicine Clinical Trial Registry ITMCTR2023000054; https://tinyurl.com/yc353hes

**International Registered Report Identifier (IRRID):**

DERR1-10.2196/55662

## Introduction

Chemotherapy-induced neutropenia (CIN) is a common complication and represents the most severe hematological toxicity associated with cancer chemotherapy. This condition has several complications, including severe infections, aggressive hospital management, life-threatening morbidity, and mortality [1]. Briefly, CIN is generally characterized by a decreased absolute neutrophil count (ANC) of <2000 cells/mm^3^ in the peripheral blood and is classified into 4 grades according to the National Cancer Institute Common Toxicity Criteria. The classifications of CIN severity are as follows: (1) grade 1 with an ANC of 1500-2000 cells/mm^3^, (2) grade 2 with an ANC of 1000-1500 cells/mm^3^, (3) grade 3 with an ANC of 500-1000 cells/mm^3^, and (4) grade 4 with an ANC <500 cells/mm^3^. The current standard treatment for CIN is the use of granulocyte colony-stimulating factor (G-CSF) to attenuate white blood cell (WBC) count and ANC [2]. The use of prophylactic G-CSF also improves patients’ quality of life (QoL) [3].

Breast cancer, particularly triple-negative breast cancer (TNBC), encompasses a heterogeneous group of cancer cells, and its treatment remains challenging. In patients with neoadjuvant chemotherapy (NAC)–treated TNBC with residual disease, higher stromal tumor–infiltrating lymphocytes (sTILs) in the resected tumor conferred an improved prognosis [4]. Moreover, higher pre-NAC sTILs and elevated pre-NAC expression of cytotoxic T-cell markers and cytokines are associated with better pathological complete response and overall survival rates [5]. sTILs, with a published cutoff of 30%, are the most widely studied marker of antitumor immunity. Besides, these sTILs could be used to predict improved response to NAC and better prognosis in the context of residual disease of patients lacking pathologic complete response. However, the mechanisms underlying the immunomodulatory effects of chemotherapy on sTILs and the influence of chemotherapy on the tumor-immune microenvironment are poorly understood.

NAC alters both tumor-infiltrating and peripheral immune cells in patients with breast cancer. The reduced levels of signal transducer and activator of transcription 1 (STAT1), activator protein-1 transcription factor subunit (Jun), and nuclear factor kappa B (NF-κB) in B cells, cytotoxic T cells, and natural killer (NK) cells, respectively, from the pretreatment stage to the mid- and posttreatment stages indicated that NAC could inhibit the activation, proliferation, and differentiation of these cells. Moreover, B cell signatures detected using single-cell RNA sequencing are significantly associated with improved survival in patients with breast cancer [6]. However, a significant decrease in the fraction of B cells and C-X-C chemokine receptor type 4, which are involved in maintaining B cell population and function, was reported during and after NAC in peripheral blood mononuclear cells (PBMCs). A previous study also demonstrated that decreased neutrophil levels correlated with a poor response to NAC [7]. High levels of systemic cluster of differentiation 8 (CD8+) cytotoxic T cells are associated with improved survival in patients with metastatic breast cancer [8]. Furthermore, the expression of cytotoxic genes in PBMCs, as opposed to tumor-immune microenvironment, may be invasive biomarkers of persistent micrometastatic disease, ultimately leading to recurrence [9]. Accordingly, lower neutrophil density in patients with breast cancer is associated with metastasis or poor prognosis.

In addition, the percentages of CD3+CD4+CD25+CD127-FoxP3+ regulatory T cells, intermediate monocytes (CD14++CD16+), and HLA-DR-CD11b+CD33+CD15+ myeloid-derived suppressor cells were reported to be significantly higher in the PBMCs of patients with breast cancer. After a single round of NAC, the intensity of CD56 expression in NK cells was significantly increased, as was the percentage of activating receptors NKp44, NKp30, and 2B4 and inhibitory receptors leukocyte-associated immunoglobulin-like receptor (LAIR) and NKG2A. The activity, rather than the proportion of NK cells, is affected [10].

In addition to conventional therapy, an increasing number of patients with breast cancer are seeking Chinese herbal medicine (CHM), as indicated by web-based surveys exploring the perspectives of patients with breast cancer concerning complementary and alternative medicine. CHM has been reported as one of the most common complementary treatments for patients with breast cancer, associated with a possible reduction in the risk of complications, including alopecia [11], neuropathy [12], and fatigue [13], as well as with an improvement in the overall QoL [14]. Moreover, the results based on claims data suggest that using CHM in combination with conventional therapy may improve the overall survival rate by 45% [15]. However, whether CHM as a complementary therapy can alleviate myelosuppression and neutropenia remains controversial. Previous studies have demonstrated that *shenqi fuzheng* injection [16] and *xihuang* pill or capsule [17] could regulate immunity and reduce tumor markers. Nevertheless, other placebo-controlled randomized controlled trials have stated that using CHM as a complementary therapy did not prevent myelosuppression in patients with breast cancer treated with adjuvant chemotherapy [18].

Understanding how NAC reshapes antitumor immunity, both in the tumor and peripheral compartments, is essential; however, the immunomodulatory effects of NAC are still unclear. The Chinese herbs frequently used in cancer treatment, including *huang qi, dang gui, huang qin, bai zhu*, and *nuzhenzi* (Table 1), could regulate immunity, but how they affect immune cell profiles is mainly unknown. Given that some CHM has been suggested to have immune regulatory effects, we hypothesized that CHM, as a supplementary therapy during chemotherapy, could lower the risk of neutropenia in patients with breast cancer. Accordingly, we are conducting a real-world pragmatic clinical trial to evaluate the effectiveness of CHM as a complementary treatment for neutropenia prevention and immune modulation during chemotherapy in patients with breast cancer.

**Table 1:** Candidate Chinese herbal medicines in this study.

Pharmaceutical Latin	Chinese name	Applications or effects	Possible side effects
*Astragalus membranaceus*	*Huang qi*	Enhance immune system, heart function, and blood sugar control	Rash, itching, runny nose, nausea, and diarrhea
*Angelica sinensis*	*Dang gui*	Heart diseases, menopausal and menstrual symptoms, high blood pressure, and inflammation	Fatigue, allergic reactions, and should not be prescribed to pregnant women and children
*Scutellaria baicalensis Georgi*	*Huang qin*	Diarrhea, insomnia, dysentery, high blood pressure, hemorrhaging, respiratory infections, and inflammation	Gastric discomfort, nausea and vomiting, and diuresis
*Atractylodes* *macrocephala*	*Bai zhu*	Antiulcer, enhance immunity, and antioxidant	Decrease blood pressure and hypoglycemia
*Fructus ligustri lucidi*	*Nuzhenzi*	Optic neuritis, leukopenia, chronic hepatitis, hyperlipidemia, coronary heart disease, and hypertension	Dizziness and diarrhea

## Methods

### Participant Recruitment

Participants are being recruited from the Hualien Tzu Chi Hospital. Recruitment began on September 12, 2021, and is expected to continue until December 2024. We expect to complete the study in the middle of 2024.

### Ethical Considerations

This study was approved by the research ethics committee of Hualien Tzu Chi Hospital (IRB 110-168-A), and informed consent will be collected from all study participants. The information of patients will be deidentified, and each visit will be compensated for US $16. This study was registered on the International Traditional Medicine Clinical Trial Registry website (ITMCTR2023000054).

### Sample Size Estimation

The sample size calculation was based on the mean and SD of the amount of CD4+ between control and treatment groups based on the published literature [19]. By setting the effect size as 0.5, the sample size of each group was estimated as 64 to achieve a power of 0.80 at a significance level of .05 using G*Power software (version 3.1; Heinrich-Heine-Universität Düsseldorf). After accounting for an anticipated dropout rate of 10%, the final sample size was determined to be 70 in each group.

### Inclusion Criteria

Patients aged 20 years and older, with a histologically confirmed diagnosis of breast cancer before the initiation of chemotherapy, are considered eligible for this study.

### Exclusion Criteria

Patients who will not be able to complete the chemotherapy and follow-up procedures based on oncologists’ experience before grouping, those who receive other medication, and those with an Eastern Cooperative Oncology Group score of 3-4 will be excluded from the study.

### Study Design

**Figure 1:**
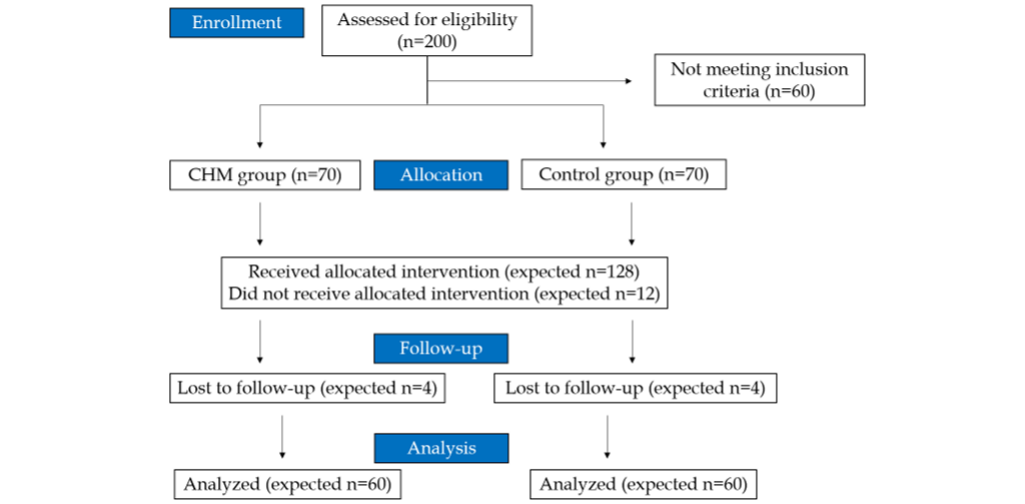
Flowchart of the study design from enrollment to analysis. CHM: Chinese herbal medicine.

This study is a real-world pragmatic clinical trial conducted at the Hualien Tzu Chi Hospital in Taiwan. Patients with breast cancer scheduled to receive chemotherapy for their cancer treatment will be recruited as per the flowchart shown in Figure 1. Patients willing to receive CHM during chemotherapy are allocated to the CHM group, whereas those in the non-CHM group will not receive CHM.

To evaluate the effectiveness of CHM, the number of WBCs, the ANC, and the use of G-CSF in patients will be measured. The following questionnaires in Taiwanese will be used: (1) the Brief Fatigue Inventory—Taiwanese Version, (2) the World Health Organization Quality of Life-BREF, (3) the National Cancer Institute-Common Terminology Criteria for Adverse Events & Patient Reported Outcomes, and (4) the body constitution questionnaire. In addition, blood samples will be collected and analyzed for immune profiles and programmed cell death protein 1 (PD-1) expression. Generally, visit 1 represents the first collection of questionnaires and blood samples before the first chemotherapy session. Following the chemotherapy regimen, the first 4 rounds of chemotherapy will be performed every 3 weeks with 3 visits (visits 2-4). Subsequently, 12 rounds of chemotherapy will be performed once per week with another 4 visits (visits 5-8, 3 weeks per visit). Patients will be followed up within 1 month of undergoing chemotherapy. Patients in the CHM group will receive complementary CHM treatment after the first chemotherapy session.

The clinical data will be extracted from the medical history system. The questionnaires will be coded and transformed into numerical values (eg, 5=very satisfied or none, 4=satisfied or light, 3=no change or intermediate, 2=unsatisfied or severe, and 1=very unsatisfied or none) to facilitate statistical analysis. Immune profiles will be analyzed using flow cytometry. The data monitoring committee, clinical trial center of Hualien Tzu Chi Hospital, is independent from the funder and competing interests. The SPIRIT (Standard Protocol Items: Recommendation for Interventional Trials) 2013 checklist is provided in Multimedia Appendix 1 [20].

### Chemotherapy and CHM Administration Protocol

**Figure 2:**
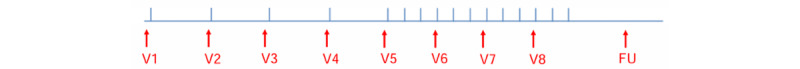
Chemotherapy, Chinese herbal medicine intervention, and data collection procedures. The questionnaire and blood collection before chemotherapy represent V1 (red arrow). The first 4 rounds of chemotherapy will be performed every 3 weeks (V2 to V4), 1 day before chemotherapy treatment. The subsequent 12 rounds of chemotherapy will be performed every week (V5 to V8), 1 day before chemotherapy treatment. The patients will be followed up (FU) after chemotherapy within 1 month. V: visit.

Upon the diagnosis of breast cancer in a patient, a clinical nurse manages the case. The questionnaire and blood collection before chemotherapy are represented by visit 1 (red arrow in Figure 2). The first 4 rounds of chemotherapy will be performed every 3 weeks, and visits 2-4 will be located in the second, third, and fifth rounds, respectively, 1 day before chemotherapy treatment. The following 12 rounds of chemotherapy will be performed every week, and visits 5-8 will be located in the first, fourth, seventh, and tenth rounds, respectively, 1 day before chemotherapy treatment. The patients will be followed up within 1 month after chemotherapy (Figure 2 and Table 2). Patients receiving CHM 3 times per day started the first round of chemotherapy.

**Table 2:** Recommended content for the schedule according to the SPIRIT (Standard Protocol Items: Recommendation for Interventional Trials) 2013 guidelines.

Time point		Enrollment	Allocation	Treatment									Follow-up
		Week 1	Week 0	Visit 1	Visit 2	Visit 3	Visit 4	Visit 5		Visit 6	Visit 7	Visit 8	
**Enrollment**													
	Eligibility screen	✓											
	Informed consent	✓											
	Allocation		✓										
**Intervention**													
	Control group			✓	✓	✓	✓	✓	✓	✓	✓	✓	
	CHM^a^ group			✓	✓	✓	✓	✓	✓	✓	✓	✓	
**Assessment**													
	ANC^b^ or WBC^c^			✓	✓	✓	✓	✓	✓	✓	✓	✓	
	Frequency of G-CSF^d^ injection			✓	✓	✓	✓	✓	✓	✓	✓	✓	
	Questionnaire			✓	✓	✓	✓	✓	✓	✓	✓	✓	
	Immune cell profile			✓	✓	✓	✓	✓	✓	✓	✓	✓	
	Adverse events			✓	✓	✓	✓	✓	✓	✓	✓	✓	

^a^CHM: Chinese herbal medicine.

^b^ANC: absolute neutrophil count.

^c^WBC: white blood cell.

^d^G-CSF: granulocyte colony-stimulating factor.

### Primary Outcome

The primary outcome used in this study is the number of immune cells, including CD4+ and CD8+ T cells (under CD45+ and CD3+ population), CD56+ NK cells (under CD45+ population), CD19+ B cells (under CD45+ population), CD14+ monocytes (under CD45+ population), and CD11c dendritic cells (under CD45+ population), as well as PD-1 expression levels in these cells. For NK cells, the expression of NKG2D will be also confirmed. The analytic panels used are listed in Table 3.

**Table 3:** Antibodies used for flow cytometry analysis^a^.

Cells or conjugate	FITC^b^	PE^c^	APC^d^	APC-Cy7^e^	PE-Cy7^f^
T cells	CD45^g^	CD4	CD8	CD3	PD-1^h^
B and NK^i^ cells	CD45	NKG2D	CD56	CD19	PD-1
Monocytes or DCs^j^	CD45	CD14	CD11c	—^k^	PD-1

^a^CD45 (304006), CD4 (317410), CD8 (301014), CD3 (344818), and CD19 (302218) were purchased from Biolegend. PD-1 (25-9969-42), CD56 (17-0567-42), NKG2D (12-5878-42), CD14 (12-0149-42), and CD11c (17-0114-82) were purchased from eBioscience.

^b^FITC: fluorescein isothiocyanate.

^c^PE: phycoerythrin.

^d^APC: allophycocyanin.

^e^APC-Cy7: allophycocyanin-cyanine 7.

^f^PE-Cy7: phycoerythrin-cyanine 7.

^g^CD: cluster of differentiation.

^h^PD-1: programmed cell death protein 1.

^i^NK: natural killer.

^j^DC: dendritic cell.

^k^Not available.

### Secondary Outcome

Secondary outcomes include the number of WBCs, the ANC, evaluation of the Brief Fatigue Inventory—Taiwanese Version, World Health Organization Quality of Life-BREF, National Cancer Institute-Common Terminology Criteria for Adverse Events & Patient Reported Outcomes, body constitution questionnaire, and frequency of G-CSF inoculation.

### Safety Assessments

All adverse events (AEs) will be recorded throughout the study. In cases where AEs are caused by chemotherapy or CHM intervention, prompt measures will be taken to address those AEs. If needed, participants will be withdrawn from the study. Continuous monitoring of the participant’s blood, liver, and kidney functions, body weight, and vital signs will be performed throughout the study. The possible side effects associated with CHM are listed in Table 1.

### Flow Cytometry Analysis

Peripheral blood (10 mL) will be collected in ethylenediaminetetraacetic acid–coated tubes and processed within 24 hours of collection. Whole blood (100 µL) will be mixed directly with the fluorescence-conjugated antibodies, including CD45, CD3, CD4, CD8, CD19, CD56, NKG2D, CD14, CD11c, and PD-1 (Table 3), protected from the light, and incubated for 20 minutes at 4 ℃. Blood cells will be washed twice with 2 mL staining buffer (phosphate buffered saline+2% fetal bovine serum), centrifuged at 300×g for 5 minutes at room temperature, and further incubated with Red Blood Cell Lysis Buffer (Thermo Fisher Scientific) for lysing the erythrocytes for 10 minutes at room temperature. This step may repeat until the red blood cell is completely lyzed. The cells will be further fixed in 2% paraformaldehyde for 10 minutes at room temperature. Subsequently, the cells will be subjected to 2 additional washes with 2 mL staining buffer and centrifuged 300×g for 5 minutes at room temperature. Then, the cells will be preserved at 4 ℃ and analyzed using flow cytometry (Lyric, BD) within 3 days.

### Statistical Analysis

All statistical analyses will be performed using the SPSS software (version 13.0; IBM Corp) or SAS software (version 9.4; SAS Institute Inc). Continuous variables will be reported as mean and SD, whereas categorical variables will be reported as frequencies and percentages. The Shapiro-Wilk test will be used to analyze the normality of baseline characteristics and outcome variables. The Mann-Whitney *U* test will be used to compare nonnormally distributed continuous data, and the 2-tailed *t* test will be used to compare normally distributed continuous data. The chi-square test will be used to compare categorical data between the groups. Both primary and secondary outcomes involve repeated measurement data. The Friedman test and repeated measures ANOVA will be used to compare the differences between every visit in each group in nonparametric and parametric data, respectively. In addition, we will use generalized estimating equations or repeated measures ANOVA to compare the differences between the 2 groups. We will evaluate the risks of each clinical characteristic by the multivariate logistic regression analysis and adjust the age and other factors or characteristics that may have bias in the analysis. We set *P*<.05 as a significant difference. All data will be recorded in a case report form.

## Results

This study was approved by the research ethics committee of Hualien Tzu Chi Hospital (IRB 110-168-A). The enrollment process began in September 2021 and will stop in December 2024. A total of 140 patients will be recruited. Data cleaning and analysis are expected to finish in the middle of 2025.

## Discussion

### Advantages and Disadvantages of Selected CHM

Traditional Chinese medicine is the most commonly used drug in Taiwan and shows significant potential in alleviating side effects related to anticancer treatments, such as chemotherapy. Even with sourcing from traditional Chinese medicine pharmaceutical factories in Taiwan certified under the Good Manufacturing Practices guidelines, our rigorous approach involves continuous toxicity monitoring throughout the study to minimize AEs.

Astragalus polysaccharide (APs), a bioactive extract of *Astragalus membranaceus*, has many biological activities, including anti-inflammatory, antioxidant, and immunoregulatory properties [21,22]. APs may modulate immunity by activating the toll-like receptor 4 (TLR4)–mediated myeloid differentiation primary response 88 (MyD88)–dependent signaling pathway [22] and induce apoptosis in human hepatocellular carcinoma cells by decreasing the expression of Notch1 [23]. Furthermore, APs inhibit TNBC cell invasion and proliferation and induce apoptosis through the PIK3CG/AKT/BCL2 pathway [24]. APs could activate macrophages to release nitric oxide and tumor necrosis factor alpha, directly blocking breast cancer cell growth [25]. *A membranaceus* extract has also been reported to inhibit breast cancer cell proliferation via the PI3K/AKT/mTOR signaling pathway [26].

Polysaccharides of *Angelica sinensis (Oliv) Diels* promote apoptosis in breast cancer cells via cyclic adenosine monophosphate response–binding protein (CREB)–regulated caspase-3 activation [27]. In a population-based case-control study in Taiwan, the use of *A sinensis* showed a significant protective effect on breast cancer (adjusted odds ratio 0.95, 95% CI 0.93-0.98) [28].

Baicalin, a flavonoid compound isolated from the roots of *Scutellaria lateriflora* *Georgi*, enhanced the chemosensitivity of breast cancer cells to doxorubicin via the upregulation of oxidative stress–mediated mitochondria-dependent apoptosis [29] and inhibited the metastasis and epithelial-to-mesenchymal transition of highly aggressive breast cancer cells by targeting β-catenin signaling [30].

Atractylenolide-I, a major bioactive component from *Atractylodes macrocephala*, suppressed tumorigenesis of breast cancer by inhibiting TLR4-mediated NF-κB signaling pathway [31] and sensitized TNBC cells to paclitaxel by blocking connective tissue growth factor expression [32].

*Fructus ligustri lucidi* could enhance chemosensitivity and induce apoptosis via Tbx3 suppression in human colorectal carcinoma cells [33]. Besides, it has been reported to induce apoptosis in glioma cells in vitro and in vivo through the regulation of the AKT/mTOR pathway [34]. In addition, it induces senescence and apoptosis in hepatocellular carcinoma cells by upregulating p21 [35].

While these CHMs have mostly exhibited antitumor effects, their effects on immune cells have not been fully elucidated. Hence, we aimed to investigate the effects of these CHMs as a complementary treatment on neutropenia, a common chemotherapy side effect known to damage qi and aggravate its depletion. Notably, these CHMs not only regulate qi but also modulate immune cells and their functions. Thus, this study may provide additional insights into therapeutic strategies for breast cancer, including immunotherapy.

### Strengths and Limitations

The strength of this study lies in its pragmatic trial design conducted in a clinical setting. Compared with randomized controlled trials, this study design has higher generalizability and provides evidence of its effectiveness in daily practice. This study has some limitations that may have affected the results. First, the internal validity was compromised. A pragmatic clinical trial design could achieve greater generalizability of the effectiveness of CHM as a complementary treatment. To improve internal validity, the statistician analyzing the data was blinded to the group assignment and adjusted for all confounding variables during data analysis. Second, there was a lack of important clinical characteristics, such as dietary and exercise habits. Although dietary intake and moderate-intensity aerobic exercise are crucial for patients with cancer during chemotherapy [36], we could not control daily diet and exercise intensity in this real-world clinical trial. In our clinical setting, oncology nurses educate all participants about the importance of healthy dietary intake and exercise habits during chemotherapy. Third, due to financial considerations, the observation time was not long enough to detect the change in the lymphocyte levels after completing chemotherapy, and further long-term and large-scale studies are needed to evaluate the variation in the number of WBCs and the ANC during the recovery phase after chemotherapy.

### Conclusions

In conclusion, we have proposed an open-label, single-center, pragmatic clinical trial and a real-world study to evaluate the effectiveness and safety of CHMs for treating CIN in patients with breast cancer. Using this study platform, we will analyze the effects of CHM on the regulation of QoL by evaluating the complications of chemotherapy and immune cell profiles. The findings of this study may contribute to developing an effective intervention for chemotherapy-related neutropenia in patients with breast cancer in clinical practice.

## Appendix

Multimedia Appendix 1 SPIRIT (Standard Protocol Items: Recommendation for Interventional Trials) checklist.

## Abbreviations

AE: adverse event
ANC: absolute neutrophil count
AP: Astragalus polysaccharide
CD8: cluster of differentiation 8
CHM: Chinese herbal medicine
CIN: chemotherapy-induced neutropenia
CREB: cyclic adenosine monophosphate response–binding protein
G-CSF: granulocyte colony-stimulating factor
LAIR: leukocyte-associated immunoglobulin-like receptor
MyD88: myeloid differentiation primary response 88
NAC: neoadjuvant chemotherapy
NF-κB: nuclear factor kappa B
NK: natural killer
PBMC: peripheral blood mononuclear cell
PD-1: programmed cell death protein 1
QoL: quality of life
SPIRIT: Standard Protocol Items: Recommendation for Interventional Trials
STAT1: signal transducer and activator of transcription 1
sTIL: stromal tumor–infiltrating lymphocyte
TLR4: toll-like receptor 4
TNBC: triple-negative breast cancer
WBC: white blood cell
